# Conceptual model for dietary behaviour change at household level: a ‘best-fit’ qualitative study using primary data

**DOI:** 10.1186/1471-2458-14-574

**Published:** 2014-06-09

**Authors:** Meena Daivadanam, Rolf Wahlström, TK Sundari Ravindran, KR Thankappan, Mala Ramanathan

**Affiliations:** 1 Achutha Menon Centre for Health Science Studies, Sree Chitra Tirunal Institute for Medical Sciences and Technology, Thiruvananthapuram 695011, India; 2 Department of Public Health Sciences (Global Health), Karolinska Institutet, Tomtebodavagen 18A, 171 77, Stockholm, Sweden

**Keywords:** Conceptual model, Framework analysis, Household decision-making, Health behaviour theories, Trans-theoretical model, Health belief model, Theory of planned behaviour, Kerala, India

## Abstract

**Background:**

Interventions having a strong theoretical basis are more efficacious, providing a strong argument for incorporating theory into intervention planning. The objective of this study was to develop a conceptual model to facilitate the planning of dietary intervention strategies at the household level in rural Kerala.

**Methods:**

Three focus group discussions and 17 individual interviews were conducted among men and women, aged between 23 and 75 years. An interview guide facilitated the process to understand: 1) feasibility and acceptability of a proposed dietary behaviour change intervention; 2) beliefs about foods, particularly fruits and vegetables; 3) decision-making in households with reference to food choices and access; and 4) to gain insights into the kind of intervention strategies that may be practical at community and household level. The data were analysed using a modified form of qualitative framework analysis, which combined both deductive and inductive reasoning. *A priori* themes were identified from relevant behaviour change theories using construct definitions, and used to index the meaning units identified from the primary qualitative data. In addition, new themes emerging from the data were included. The associations between the themes were mapped into four main factors and its components, which contributed to construction of the conceptual model.

**Results:**

Thirteen of the *a priori* themes from three behaviour change theories (Trans-theoretical model, Health Belief model and Theory of Planned Behaviour) were confirmed or slightly modified, while four new themes emerged from the data. The conceptual model had four main factors and its components: impact factors (decisional balance, risk perception, attitude); change processes (action-oriented, cognitive); background factors (personal modifiers, societal norms); and overarching factors (accessibility, perceived needs and preferences), built around a three-stage change spiral (pre-contemplation, intention, action). Decisional balance was the strongest in terms of impacting the process of behaviour change, while household efficacy and perceived household cooperation were identified as ‘markers’ for stages-of-change at the household level.

**Conclusions:**

This type of framework analysis made it possible to develop a conceptual model that could facilitate the design of intervention strategies to aid a household-level dietary behaviour change process.

## Background

Intervention planning related to health is a complex process in any setting. Health behaviour theories (HBT) help us to gain an understanding of behaviour and its many determinants and often form the basis of developing interventions [1]. It has also been shown that interventions having a strong theoretical basis are more efficacious; providing a strong argument for incorporating theory into the planning and development of health interventions [2].

HBT have often been used in their original form, and at times with modifications or in combination with other theories [2]. However, most of the HBTs originated in the Western world and were originally proposed for a specific behaviour in a specific context [3-5]. Many have subsequently been validated or used in relation to other behaviours [5-7]. More importantly, all these theories primarily focus at the individual level [1]; and, consequently, intervention studies based on HBT, also focus on the behaviour change of individuals [8]. The most commonly used of these HBTs are the trans-theoretical model, the Health Belief model, the Social Cognitive model and the Theory of Planned Behaviour [1,2]. There is a dearth of behaviour theories related to community or family-level behaviours, in spite of the amount of community-based health interventions being planned and carried out today [2]. A few models like the social ecological model have been developed in an attempt to explain the health behaviours in terms of interactions at the various levels in a community, but these have not really progressed to the stage of theory building [9].

During the planning stages of a dietary behavioural intervention for rural households in Kerala, India, we identified two major gaps: 1) none of the HBTs was developed or tested sufficiently in a setting like India; and 2) all the HBTs and most dietary behavioural interventions targeted the individual, while the dietary decision-making process in this setting essentially took place at the household level [3, Daivadanam et al: personal communication 2014]. Hence our interventions should be targeted at the household and not the individual. One way of overcoming these gaps was to develop a conceptual model, defined as “a network of interlinked concepts that together provide a comprehensive understanding of a phenomenon or phenomena” [10] (p. 51). Conceptual models are typically based on or guided by theory and grounded in reality to make it directly applicable for the context and setting being studied. This has the distinct advantage of being able to incorporate theory with other factors that have a bearing on the unique aspects of this specific situation. Here, the phenomenon under study was the dynamics of a household-level behaviour change process; and the unique factors were: the household being the target of intervention; the context and culture; and the practice of a collective as opposed to individual decision-making [11].

Therefore, the objectives of this study was to identify relevant theories of behaviour change and develop a conceptual model based on these theories that could facilitate the planning of intervention strategies to aid the dietary behaviour change process at the household level in rural Kerala.

## Methods

### Study setting and participants

We chose a qualitative approach to achieve the objectives and the study has been described here based on RATS guidelines for reporting qualitative studies [12]. The study was a part of the exploratory phase for a community-based dietary behaviour change intervention for prevention of chronic non-communicable diseases in rural Kerala [11]. It was conducted in Thiruvananthapuram district with a population of about 3.3 million. Kerala follows a de-centralised system of administration at the state, district and block panchayat-levels. The latter are further de-centralised to rural (grama panchayat) and urban (municipality) administrative units [13]. *Chirayinkeezhu taluk*, with four block panchayats, in turn consisting of 22 grama panchayats and 2 municipality areas, is the setting for the study. It is one of four revenue divisions of Thiruvananthapuram district and has a population of 550 thousand (about 130 thousand households). The study was conducted in the rural areas in two of the grama panchayats. One coastal and one non-coastal area were selected, as fishing was the predominant occupation along the coast and seafood formed a major part of their diet in contrast to the non-coastal area. Kerala has a well-developed and functioning women’s self help group network called the *Kudumbasree*, which is organised in the form of neighbourhood groups or *ayalkootams*. Different socio-economic areas were identified through the Kudumbasree registers based on their household income, and the individual households were sampled purposively from these localities. With the help of community volunteers, the participants were invited for the focus group discussions and individual interviews.

We invited men and women of different religions and socio-economic strata (SES), between 24 and 75 years of age to participate in three focus group discussions (FGDs) and 17 individual interviews. The participants of FGD 1 and 3 were from low and middle SES respectively; while FGD 2 included participants from both low and middle SES. The individual interview participants however, belonged to different SES (3 individuals from low SES; 8 from middle; 6 from high). As the study focused on dietary decision-makers, the participants were mostly female head of the households and any other member identified by her as being involved in dietary decision-making in their household. The voices of the men in the community were also found to be important, and the third FGD was primarily organized among men.

### The research team

The research team included the principal investigator (MD), an assistant, one Swedish (RW) and three Indian (MR, TKSR and KRT) public health scientists. The first author (MD), who was also the interviewer and moderator is a medical practitioner with public health training. MR is a qualitative researcher with significant experience in framework analysis. TKSR and RW are also well experienced in qualitative research and looked at the data with insider and outsider perspectives respectively. KRT being a medical and public health practitioner in the state also provided an insider perspective.

### Data collection procedure

The community volunteers who arranged the FGDs and interviews, resided in these areas and interacted with the people on a regular basis. They approached the residents and requested time for the respective events. None of those who were approached with a request to participate declined the invitation. All individual interviews were conducted in the residences of the participants, except one, which was conducted in a neighbour’s house. FGDs 1 and 2 were conducted in a local school, while FGD 3 was conducted in the residence of one of the participants in the coastal village. Both the interviews and FGDs were conducted based on an interview guide to understand: 1) feasibility and acceptability of a proposed dietary behaviour change intervention; 2) beliefs about foods, particularly fruits and vegetables; 3) decision-making in households with reference to food choices and access; and 4) to gain insights into the kind of intervention strategies that may be practical at community and household level (Additional file 1). The individual interviews took between 45 minutes to 1.5 hours, while the FGDs went on longer, between 1.5 to 2 hours. Data collection was stopped once we reached saturation.

### Data analysis

All of the collected data was used for the analysis. Parts of the same data (section I-IV, Additional file 1) have been analyzed using qualitative content analysis in another study by the authors to understand the process of food decision-making in rural households [Daivadanam et al: personal communication 2014]. The objective of the present study was to develop a conceptual model that was grounded in the reality of everyday living, in order to facilitate the development of dietary intervention strategies for a household-level behaviour change process. To this end, the data was analysed with a view to identify how this change process can be facilitated by understanding current practices and their reasons, how and why changes are triggered in households and how these changes were dealt with.

The data were analysed using a modified framework analysis [14] similar to the innovative ‘best-fit’ framework synthesis methodology used for systematic reviews of qualitative literature [15,16]. Here, we worked on primary qualitative data using a combination of inductive and deductive reasoning. Unlike the framework analysis, which is primarily a deductive approach [14,17], we did not try to ‘fit’ all the data into pre-identified or *a priori* themes.

The four steps involved in the data analysis process are outlined below:

Step 1: *Identification of relevant behaviour change theories and ‘a priori’ themes*: Four behaviour-change theories were identified from literature as relevant to the context [1,2]: one change theory - the Trans-theoretical Model (TTM) [4]; and three explanatory theories - Health Belief Model (HBM) [18], Social Cognitive Model [5], and Theory of Planned Behaviour (TPB) [19]. All core and peripheral constructs of the four theories were listed and defined using, as far as possible, definitions of the original authors (Table 1).

**Table 1:** **Constructs identified from relevant behaviour change theories with the corresponding *a priori *themes**

**Theory and construct**	**Identified *a priori *theme**
*Theory I: Trans-theoretical or Stages-of-change model for behaviour change*	
• Stage construct	1. Identifying stages-of-change
	This was considered as one theme with the focus on identifying cues to differentiate households to three stages-of-change, instead of the original five. Hence for this study:
	• Pre-contemplation = Pre-contemplation
	• Intention = Contemplation + Preparation
	• Action = Action + Maintenance
• Decisional balance	2. Perceived pros and cons
• Self-efficacy	3. Self-efficacy
• Change processes	4. Awareness
	5. Emotional reaction
	6. Effect of behavior on others
	7. Social alternatives for disadvantaged
	8. Self-evaluation
	9. Identifying temptations
	10. Helpful relationship
	11. Substitution
	12. Reinforcement or rewards
	13. Commitment
*Theory II: Health Belief Model*	
• Perceived susceptibility	14. Perceived susceptibility
• Perceived benefits	15. Perceived benefits
• Perceived barriers	16. Perceived barriers
• Perceived seriousness	17. Perceived seriousness
• Cues to action	18. Perceived facilitators
• Modifying variables	19. Personal modifiers
• Self efficacy	3. Self-efficacy*
*Theory III: Theory of Planned Behaviour*	
• Attitude (towards the behaviour)	20. Attitude
• Subjective norms	21. Subjective norms
• Perceived behavioural control	3. Self-efficacy*
*Theory IV: Social Cognitive Model*	
• Knowledge	4. Awareness*
• Perceived self-efficacy	3. Self-efficacy*
• Outcome expectations	15. Perceived benefits*
• Goals	22. Goal setting
• Perceived facilitators and impediments	18. Perceived facilitators*
	16. Perceived barriers*

*Constructs from different health behaviour theories having similar definitions are identified as one *a priori* theme.

Step 2: *Deductive analysis process: finalizing ‘a priori’ themes*: The deductive part of the analysis was conducted in two stages. In the first stage, the data was fragmented into meaning units and the identified *a priori* themes were initially applied to five transcripts (2 FGDs and 3 individual interviews). Those *a priori* themes that did not fit the data at this stage were excluded and the remaining were finalised as such or with modifications. In the second stage, the finalized *a priori* themes were then re-applied across all the transcripts and used to index the meaning units identified from the data.

Step 3: *Inductive analysis process: identifying and finalizing ‘new’ themes*: Data not ‘fitting’ into these *a priori* themes were not ‘squeezed’ into them; rather we identified newly emerging themes that were relevant to the setting [16], but were not part of the core or peripheral constructs identified from the original behaviour change theories.

Step 4: *Mapping the results: finalizing the factors in the conceptual model*: Once all the themes were finalized, associations between them were explored, and they were mapped into main, component and sub-factors. A visual representation of the conceptual model was also developed to emphasise its dynamic nature.

### Ethical considerations

This study was conducted according to the guidelines laid down by the Indian Council of Medical Research. The Institutional Ethics Committee of Sree Chitra Tirunal Institute for Medical Sciences and Technology, Thiruvananthapuram, Kerala, India approved all the procedures involving the study participants as well as the interview and FGD guidelines. Participants were recruited only after recording verbal informed consent.

## Results

### Relevant behaviour change theories and indexed *‘a priori’* and new themes

Twenty-two constructs were identified as *a priori* themes from four HBTs (Table 1). Three of these HBTs (TTM, HBM and TPB) were assessed as relevant for the conceptual model after step 2. All core constructs of SCM, excluding goal setting, were common with the other three theories. Goal setting was also excluded in step 2, as it was not found to fit, when the *a priori* themes were applied across the five initial transcripts. Hence, the SCM was excluded after step 2 of the analysis.

The status of the twenty-two *a priori* themes after the first stage of deductive analysis process is described in Table 2. Seven of the themes were not found to be relevant and excluded and six were retained as such, while nine were retained with modifications. Most of the modifications were minor involving change of name to better fit the context under study. ‘Perceived pros and cons’, ‘perceived benefits’ and ‘perceived barriers’, which had subtle differences were combined to form one theme called ‘decisional balance’.

**Table 2:** Finalization of themes after deductive and inductive reasoning

**Status of *a priori *themes after deductive reasoning**	
***A priori *themes retained as such**	
1.	Awareness
2.	Self-evaluation
3.	Helpful relationships
4.	Perceived seriousness
5.	Personal modifiers
6.	Attitude
***A priori *** **themes retained with modifications**	
1.	Identifying stages-of-change: the definition modified to identify only three stages: pre-contemplation, intention and action
2.	Household efficacy: modification of ‘self-efficacy’
3.	Decisional balance: combination of ‘perceived pros and cons’, ‘perceived benefits’ and ‘perceived barriers’
4.	Substitution opportunities: modification of ‘substitution’
5.	Perceived risk: modification of ‘perceived susceptibility’
6.	Perceived societal response: modification of ‘subjective norms’
7.	Cues to action: modification of ‘perceived facilitators’
**Excluded *a priori *themes**	
1.	Emotional reaction
2.	Effect of behaviour on others
3.	Social alternatives for the disadvantaged
4.	Identifying temptations
5.	Reinforcement or rewards
6.	Commitment
7.	Goal setting
**New themes identified after inductive reasoning**	
1.	Accessibility
2.	Perceived needs and preferences
3.	Societal norms
4.	Perceived household response

The stage construct was also modified to ‘identifying stages-of-change’ to look for specific beliefs and concerns that would differentiate households on the basis of the stages-of-change. Since this study was conducted as a preparation for a community-based dietary behavioural intervention, we decided to modify the original stages-of-change [4] from five to three for practical purposes. Thus, the modified stages were as follows: 1) pre-contemplation, which was the same as the original pre-contemplation; 2) intention, which combined contemplation and preparation stages; and 3) action, which combined action and maintenance stages [4]. We were also concurrently developing a simple algorithm-based questionnaire as a tool to identify the household-level stage-of-change that could be administered by community volunteers. So, during this study, our focus regarding staging was to identify beliefs, concerns or thoughts that could be used to identify the different stages.

Four new themes: accessibility, perceived needs and preferences, societal norms, and perceived household response were identified using inductive reasoning from data that did not fit into any of the *a priori* themes (Table 2). Accessibility, in particular its affordability aspect was found to be important in the lower SES. Similarly, the importance given to perceived needs and preferences in households were found to differ based on the position or expected role of the individual in the household hierarchy.

The final themes that were mapped to construct the conceptual model are defined in Additional file 2.

### Mapping the results: factors in the conceptual model

The final step of the modified framework analysis was the mapping of the final themes to identify the main factors, component factors and sub-factors of the conceptual model based on the research objectives (Table 3). During the analysis, the sub-factors were further divided into categories, but these are not shown in Table 3. In this section, we have mainly described each of the main and component factors; and outlined any associations that were identified. All quotations are in *italics.* Any text within the quotes that are enclosed by square brackets have been inserted by the authors.

**Table 3:** Mapping the results of the best-fit framework analysis to identify the main, component and sub-factors of the conceptual model

**Main factors**	**Component factors**	**Sub-factors**
1. Impact factors	Decisional balance	Not worth the effort or cost
		Relative costs
		Maximize benefit, minimize cost
	Risk perception	Perceived risk
		Perceived seriousness
	Attitude	Contrasting attitudes: contrasting behaviours
		Value-laden attitudes: changes perceptions and use
2. Change or facilitating processes	Action-oriented processes	Cues to action
		Substitution opportunities
		Self-evaluation
	Cognitive processes	Household efficacy
		Perceived household response
		Awareness
		Helpful relationships
		Perceived societal response
3. Background factors	Personal modifiers	Individual: Age, occupation
		Household: household composition, place of residence
	Societal norms	Stereotyped roles
		Financial dependence
4. Overarching influence	Accessibility	Affordability
		Availability
	Perceived needs and preferences	Perceived needs
		Household routines
		Specific preferences, tastes and habits

#### 1. Impact factors

Three impact factors, which were likely to have the greatest influence in making a decision to change or maintain a dietary behaviour were identified: decisional balance, risk perception and attitude.

• *Decisional balance* was the strongest and most recurring theme across all interviews and FGDs. Certain behaviours were adopted as a result of a perceived imbalance in the decisional stakes relating to costs and benefits. If something was not considered to be worth the effort or the cost involved, then it was unlikely to be pursued: attempting to cultivate on unviable land or small land holdings; or burdensome, tiring or expensive transport to purchase one pack of vegetables, when plain rice and fish or dry coconut chutney would cost far less effort and money: *“To get a pack of vegetables that cost minimum twenty rupees, we have to pay a bus fare of ten rupees. So, we need total thirty rupees [1USD ≈ 50 Indian Rupees]. Some of us have to take an auto for forty-five rupees to get this twenty-rupee pack. Often there is no bus in the morning … Sometimes, when we feel we cannot walk, we just decide that there will be no vegetables on that day.”* (FGD 1; Female; 65 years_b)

• Costs were also relative when the perceived need was highly prioritized, like those for children, which are considered to be essential and worth any cost. Similarly, healthier or easier options, like fruits and cut and packaged vegetables, were relatively more expensive, making them unattractive, when compared with cheaper alternatives, like snacks (biscuits), often portrayed in the visual media as healthy. Households also attempted to maximise use and minimise wastage or cost by purchasing ‘mixed vegetable packs’ or saving expensive fruits for children, who ‘need it more’ rather than ‘waste it’ on adults.

• *Risk perception* was different among those who had a member with a chronic disease in their family and those who did not. Those who perceived it seriously took the effort to make arrangements for dietary restrictions, sometimes, even in the face of their family’s indifference: *“If they [daughter and son-in-law] make food with too much salt, that day I only eat rice. I won’t take anything else.”* (Interview 3; Female, hypertensive; 65 years); while others were not so particular or careful in spite of medical advice: *“… I eat more than I should even though I have sugar.”* (Interview 5; Female; 44 years) Perceived risk of becoming diabetic, hypertensive or overweight was found to be higher among those with family history or if someone in their household already had the problem, and among those who self-evaluated their food habits and consumption of oil in particular, to be unhealthy: *“… If I cook, I use less oil, but if my husband or son cook, they use more oil because they like the taste … I already have sugar, he [husband] has cholesterol, only God knows what else we will get …”* (Interview 5; Female; 44 years)

• *Attitude* towards certain behaviour or foodstuffs were also found to drive behaviours. The same behaviour could have entirely different motivations based on contrasting attitudes as shown by the example of ‘eating out’. On the one hand, it was perceived as undesirable and therefore resorted to only in exceptional circumstances, such as being a caregiver to someone admitted in the hospital. On the other hand, when it was perceived as a source of ‘good food’, it was over-indulged and supported financially even in resource-poor households. This was the case of an adolescent son studying in college, who ate *‘only good food because he was eating out'.*

#### 2. Change or facilitating processes

Change or facilitating processes were of two types: action-oriented processes that guide behaviour change by instituting or advising specific actions at specific points; and cognitive processes that guide the mental process of acquiring and processing knowledge and experiences. There were three action-oriented and five cognitive processes.

• *Action-oriented processes*

• *Cues to action* relates to identifying occasions to intervene in the day-to-day working of a household, for example in relation to food preparation and procurement or access to certain foodstuffs within the household. Most households had a set routine, where certain members were responsible for either food procurement or preparation or both: *“I or my daughter cook at home. … I am the one who goes to the market.”* (Interview 7; Female; 52 years). This was also true for the procurement of certain food stuffs like oil for instance which was often milled from their coconuts: *“We don’t sell the coconuts. We have about two to three coconut trees. We use the coconut scrapings in all our curries and we save the rest … when they total to a number of fifty or hundred, we extract oil from those …”* (Interview 1; Female; 36 years)

• *Substitution opportunities* were occasions where behaviours were altered individually or collectively within households to accept the next available alternative. For example, food preparation or procurement habits were shifted in response to increased food prices: *“Since coconuts have become expensive, a lot of us have shifted to mizhukku completely. [Mizhukku is a fried vegetable preparation that does not require coconut scraping]”* (FGD 2; Female; 67 years); or to avoid undesirable consequences from adulterated or stale foods: *“… I worked in a bakery earlier…old things were often repacked and sold. So, I don’t buy cake items anymore.”* (Interview 2; Female; 63 years)

• *Self-evaluation* for food habits was more specific for dietary components. The participants recognised the gaps in their behaviour, particularly relating to the use and measurement of salt, sugar, coconut and oil: *“we use more sugar… everyone drinks lot of tea at home …”* (Interview 8; Female; 29 years)*; “When we have fresh oil, we just use it and don’t realise how much we are using.”* (FGD 2; Female; 39 years)

• *Cognitive processes*

• *Household efficacy* was a reflection of a household’s confidence to make the change: *“Children won’t like it … but, we are willing to try”* (Interview 10; Female; 24 years); to positively influence other household members: *“Reallocating our budget to buy more fruits and vegetables will not be difficult. We are the ones who tell them [men] what to buy for the children. So, if we tell them to buy something different, they will buy that.”* (Interview 14; Female; 23 years); or to consider the practicalities required to make the change: *“We can reallocate money that we spend on snacks and other things… and buy fruits…”* (Interview 2; Female; 63 years).

• *Perceived household response* was of two types: perceived willingness, and perceived cooperation of other household members to consider a dietary behaviour change intervention. Of these two, the latter was identified as a proxy for commitment at the household level. The difference between willingness and cooperation was a commitment factor in the latter regarding other household members, to make specific rather than general changes: *“Our children like to grow things… so, they will cooperate… will be interested in growing a kitchen garden*” (Interview 2; Female; 63 years).

• *Awareness* was primarily related to a lack of understanding about the dietary guidelines, benefits of local fruits and vegetables. Similarly, harms related to excess consumption, particularly sugar, was also reflected in their household practices: *“We know that it is good to take less salt for pressure and less sugar and sweet things if we have sugar [diabetes]…but, we don’t know the amounts, like how much oil or salt we can use …”* (FGD 3; Female; 62 years) Dietary changes were mainly discussed in the context of restrictions after diagnosis and not as preventive measures, though many of the FGD participants observed the occurrence of diabetes in younger individuals in the present generation: *“Aren’t these diseases hereditary? Can we really prevent any of these diseases by changing our food habits? At least if we do something like this now, will our children have a healthier life?”* (FGD 3; Male; 70 years) Misconceptions related to local fruits and vegetables, polished rice and illness-related dietary restriction were also common and they were mostly based on hearsay: “*… heard that Madhura cheera [type of locally available greens] causes cancer … notices were posted about it in another town. We had lots of it here … we stopped eating and cut everything down.”* (Interview 7; Female; 52 years). Many participants however had knowledge, that was not translated to practice; related to diet-disease links, benefits of local fruits and vegetables and dietary restrictions for diseases like diabetes and hypertension: *“Drumstick leaves are very good as it has lot of vitamins…”* (Interview 10; Female; 24 years)*; “We don’t use tapioca because it is not good for my sugar.”* (Interview 9; Female; 57 years)

• *Helpful relationships* were of two types, support from spouse and children, which was considered essential: *“I cook based on my husband’s preference…”* (Interview 14; Female; 23 years)*; “… but it will be difficult to cut bakery items and biscuits … children are not going to like it.”* (Interview 4; Female; 42 years); and the special relationship shared between women in the households because of their shared chores: *“Usually me or my daughter goes to the market … My daughter or grand-daughter does the cooking … between us women, we manage all these things”* (Interview 15; Female; 75 years)

• *Perceived societal response* in relation to ‘what other people thought’ and ‘what other people did’ was also found to have some influence, particularly the latter, where it was associated with the perceived practices of people in their neighbourhood: *“We are not interested in such a program … In this area, there are only one or two families, who buy vegetables … so, not sure if anyone will be interested …”* (Interview 13; Female; 60 years)

#### 3. Background factors

Background factors are the factors that influence the behaviours, but over which individuals or households have little control. These included personal modifiers and societal norms.

• *Personal modifiers* included individual and household level factors. Factors at the individual level included age and occupation; particularly the regularity of income and stability of the job. The household-level factors included the composition of the household in terms of children or members with diagnosed diseases; and place of residence, which decided the proximity to markets, availability of transport, size of land holdings and viability of the land for cultivation.

• *Societal norms* were equally if not more important, as they influence dietary practices from procurement to consumption through stereotyping of roles, which is a dominant feature of this setting. As a result women were expected to be responsible for food preparation and procurement and held accountable for the results in terms of taste and quality. This fostered a feeling of entitlement in men and older children, particularly sons, that their preferences be prioritized in the way food is prepared and presented: *“… fish has to be fried real well. If it’s not, he [husband] wouldn’t eat it … he also like to have his curries real hot and sour, with plenty of salt. He won’t eat it otherwise.”* (Interview 1; Female; 36 years) Societal expectations of men as breadwinners and women as home makers and the lack of jobs for uneducated women also kept them dependent on their men for financial access and brought with it a lack of entitlement for those not employed for wages: *“He [husband] works and hands over the money to me to buy things [food]. I also buy everything that the children need.”* (Interview 1; Female; 36 years). The needs of the non-earning homemaker were therefore not prioritised, even if she was the decision maker and bore the primary responsibility for both food procurement and preparation.

#### 4. Overarching influence

Accessibility and perceived needs and preferences were found to be overarching influences, with the potential to make households resistant to change unless appropriate strategies were to be employed.

• *Accessibility* included both affordability and availability of which affordability had serious implications. If affordability was relative, i.e., based on perceptions, this was reflected in the importance attributed to certain foodstuffs: *“… buy biscuits and mixture for children daily for about ten rupees… about three-hundred to five-hundred rupees every month … we don’t have the finances to buy fruits … we are not particular that we should eat fruits or vegetables every day.”* (Interview 7; Female; 52 years) However, if the affordability was basic or absolute, when even basic needs were taken care of with difficulty, then behaviour change strategies would be unlikely to have any impact if not accompanied by policy measures like subsidies: *“Even for water, we have to go by ferry and get it. We haven’t had water for two days. … If water does not come, then we have to go to Sharkara [across the backwaters] to get it. Water is more important or vegetables? We can still manage rice and fish for food, but we can’t do anything without water.” [The water they get in their wells was salty, so they were dependent on either the bore well from across the backwaters or government water supply through pipes or tankers for drinking water]* (FGD 2; Female; 41 years_a)

• *Perceived needs and preferences* were described in terms of taste, habit and household routines that have been entrenched in families for decades and have strong cultural roots as well. Participants felt that interventions that were not in line with the preferences of household members, particularly children and spouse, had little chance of success: *“…we buy [vegetables] everyday but our children don’t eat vegetables … my daughter is better, but my son doesn’t eat at all …”* (Interview 17; Female; 35 years)

### Stage identification: markers of stages-of-change at household level

A few participants were categorical in their disinterest due to reasons like financial problems and strong preferences of household members: *“This [intervention] is not going to work here … It is not possible to make such changes.”* (FGD 3; Male; 62 years). However, many participants expressed their interest to be part of a dietary intervention program or willingness to make changes, but this was not found to be relevant in differentiating households based on stages-of-change. Asking about a possible time frame for change, which is arbitrarily considered as six months or more for pre-contemplation and one month for preparation, did not elicit any differentiating responses either. So, it had to be discarded after the first few interviews. Since simple verbal expressions of interest or an arbitrary time frame were insufficient to differentiate stages, we concentrated on understanding other beliefs and concerns that could identify the stages, both during the interviews and in the analysis. Household efficacy and perceived household response, both cognitive change processes, were found to better determine the stage-of-change at household level. While it was not possible to identify the exact combination of these two that would predict a specific stage, it was nevertheless clear that household efficacy and the cooperation component of perceived household response were associated with the higher stages-of-change: intention and action.

### The conceptual model

**Figure 1:**
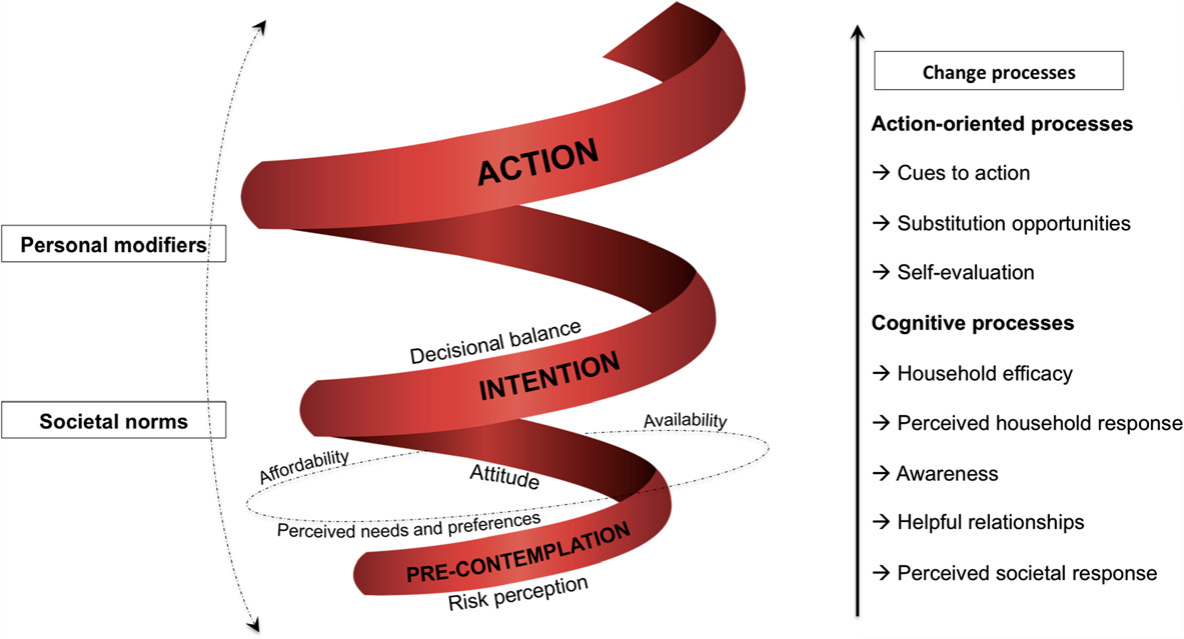
**Visual representation of the conceptual model to facilitate planning of dietary intervention at household level.** The visual representation of the conceptual model is composed of five parts: the central three-stage spiral representing the three stages-of-change; the ‘impact factors’ along the stem of the spiral; the ‘change processes’ on the right that enable progress from one stage to the other; the background factors on the left that act at all stages; and the overarching influence represented by a transverse plane between the first two stages.

The findings of the ‘best-fit’ framework analysis were assimilated and merged into a schematic representation of the conceptual model (Figure 1). Thus, the model is composed of five parts: the central three-stage spiral representing the three stages-of-change; the ‘impact factors’ along the stem of the spiral; the ‘change processes’ on the right that enable progress from one stage to the other; the background factors on the left that act at all stages; and the overarching factors represented by a transverse plane between the first two stages.

Households could therefore enter the spiral at any stage and move forward aided by the behaviour-change strategies through the change processes; or relapse if the barriers prove stronger than the facilitating factors. Risk perception, attitude and decisional balance were the three key impact factors identified through the analysis, of which the latter was the most consistent. While they can influence outcome at any level, it is more important to address these through various strategies during the first two stages when resistance seems to be maximum. Similarly, specific change processes also work best at specific stages except the stage-identifying factors, which are relevant to all stages. Hence, the change processes are represented in a hierarchical manner based primarily on how the TTM explains the interaction of various change processes at various stages [4,20]. This is supported by our analysis, which found the cognitive processes to be more relevant towards the lower stages where the resistance to change is often a psychological phenomenon. On the other hand, the action-oriented processes were more relevant towards the later stages, when the household’s response to the proposed behaviour change was more clear or ‘committed’.

In addition to the impact factors and change processes, we have identified background factors or modifiers, namely the societal and gender norms that act at all stages. The three overarching elements: affordability; availability; and perceived needs and preferences, were further identified during this analysis. These elements may act as barriers, preventing households from progressing across the stages, and as resistance is likely to be at a maximum before entry into the intention stage, they have been placed as a transverse plane between the pre-contemplation and intention stages. Basic or absolute affordability could prevent households from even entering the spiral, unless this issue is specifically addressed.

## Discussion

The main finding of this study is the conceptual model that is applicable for dietary behaviour change at household level. To the best of our knowledge, existing behaviour change models and theories focus on individuals and do not incorporate a household or collective component. We studied the dynamics of behaviour change within households and how this can be influenced and facilitated, while attempting to identify specific ‘markers’ to assess the stages-of-change at household level. This understanding was linked to the existing theoretical constructs of established health behaviour theories in order to construct the innovative conceptual model. Although these theories provide an understanding of health behaviour and the change process in general, the gaps that arise due to change of setting and context [3], needs to be addressed. We chose to do this by combining the original constructs, with modifications where necessary, to findings arising from our data. We considered this method of analysis as the best way to develop a conceptual model ‘deduced’ from existing theories, but still ‘grounded’ in data so as to make it relevant for this setting and context.

In the present study, one change theory (TTM) was combined with two explanatory theories (HBM and TPB) to develop the conceptual model. In addition, the stage construct from the TTM was modified to three instead of the original five stages for two reasons. First, our analysis revealed that two of the cognitive change processes, household efficacy and perceived household response could be used to identify the stages-of-change. The specific combination of these two that could identify a specific stage was however undertaken only in our subsequent work where we developed a tool to identify the household-level stage-of-change. Moreover, two factors in any permutation or combination can yield a maximum of only four outcomes and not five. Hence, in the interest of classifying households clearly to a specific stage-of-change, it was decided to modify the stages construct to three clearly defined stages at the household level. Second, this conceptual model was developed to inform the development of behaviour change strategies in a community-based study. The strategies were to be delivered by community volunteers and hence, it was important to develop strategies that were simple and easy to deliver. Identifying five stages would have necessitated five sets of strategies, which would have made the intervention complicated and very difficult to manage. Other studies have also used modifications of the stage construct quite successfully in the past [21,22]. Hence, the conceptual model was constructed to achieve a specific objective and intended for practical use.

### Comparison with HBTs and other behaviour change models

Many studies have modified, combined or built on existing theories, as a single theory may not address the issues related to the specific study setting [2]. For example, the COM-B system proposed by Michie et al [23], explains the interaction of capability (C), opportunity (O) and motivation (M) to generate behaviour (B) [23]. While this is highly relevant for behaviour at the individual level, it seems to be insufficient to address the same at the household level, as is the case in the present study. Here, opportunity would rest on the decisions made by someone else and an individual’s motivation and capability, however strong, may not stand against the overall collective nature of the decisions made in the household. Although the Behaviour Change Wheel model [23] provides a broad framework to develop behavioural interventions, its contextualization possibilities need further exploration. For example, factors specifically applicable to settings such as ours, where the decision-making process incorporates a collective component, may need to be added. Modifying or combining theories or models alone may therefore not address all aspects related to a specific setting, simply because these theories or models were developed in a different context with different baseline assumptions [3]. We addressed this in our study by integrating the understanding derived from theory with the findings from our primary data.

Previous work had identified that dietary decision-making processes in rural Kerala essentially took place at the household rather than an individual level [3, Daivadanam et al: personal communication 2014]. As a result some of the constructs were not found to be applicable to this process in this context and these were excluded. Four of the excluded themes: emotional reaction, identifying temptations, reinforcements or rewards and commitment are highly relevant in the individual context, but not so in the household context. They seem to disappear or get lost during the collective decision-making process. It is possible, that further studies in this area could lead to identifying proxy themes for each of these at the household level, like ‘commitment’ for example, which could not be identified at the household level. However, the category ‘perceived household cooperation’ under the sub-factor ‘perceived household response’ was identified as a potential proxy for ‘commitment’ at the household level.

Based on the contextual realities of the study setting, this model is currently focused on the household level. However, it could potentially be modified and applied to other settings and situations where a collective decision-making component is pre-dominant, such as in many other low- and middle-income settings, or in studies involving children, immigrants or other vulnerable groups in high-income countries. The discarded themes may then become important, when the model is expanded to include individual and community level factors relevant for dietary behaviour change.

### Implications: devising behaviour change strategies

• Each of the *a priori* and new themes that make up the conceptual model represent specific areas to focus on while developing intervention strategies, and these were addressed through four general and five stage-matched strategies [11].

• The stage-identifying processes were particularly important for the development of a household staging tool, which was one of the main features of the forth-coming dietary behavioural intervention.

• In addition, specific issues were also identified that needed to be tackled: the issue of addressing community awareness to improve perceived societal response; addressing misconceptions with acceptable alternatives (e.g. using locally available fruits as an alternative to buying commercially grown ones); addressing use of coconut oil using strategies that would lead to reduced consumption without increasing food-related expenditures.

• An overall household involvement in the intervention would be key to addressing and minimising the adverse impact of behavioural strategies that are not in line with stated preferences of the most influential household members. Similarly, affordability for certain foods, which was based on perception or preference, could be potentially addressed through improved awareness.

• Though the rationale for selecting a coastal and non-coastal area was primarily to ensure the inclusion of areas with predominant seafood consumption, it had major implications for the intervention that followed. The coastal and non-coastal areas also differed significantly in terms of the availability of fruits and vegetables as local vegetable markets were fewer in the coastal areas compared to the non-coastal areas. Moreover, household cultivation of common vegetables was also more difficult due to the loose sandy and salty soil. Hence, vegetables and fruits were mostly procured from markets in nearby towns, which was comparatively more expensive for the coastal households due to the higher transportation costs involved. This had a bearing on the availability and affordability of fruits and vegetables and had to be specifically addressed during the intervention.

• Finally, the findings have also had one major impact on the study design, through the understanding that the low-income households would require specific and targeted policies and subsidies at the very least [24], to enable an equitable opportunity for them to even enter the behaviour-change spiral. Hence, the intervention study focused on a more homogenous group of middle-income households [11].

### Strengths and limitations

This study has two major strengths: 1) It is an integration of existing theories with findings from primary data analysis, which has enabled us to overcome the gaps in existing theories pertaining to their focus on the individual. This has further ensured that the conceptual model is theory-based but grounded in the reality of the study setting, making it a strong base for developing intervention strategies. 2) The combined use of inductive and deductive reasoning allowed new themes to emerge, which are critical to a dietary decision-making process at the household level. This would not have been possible if we had used framework analysis in its original form [17]. Moreover, the FGDs and interviews complemented each other, with the former contributing to our understanding of general practices and common beliefs, and the latter providing deeper insights into the food decision-making process and specific procurement or consumption practices within households.

While the combination of deductive and inductive reasoning has been used before with both primary data as well as in systematic reviews of qualitative papers [14,16], it is still relatively new. This could be considered a limitation, as it is not an approach that has been ‘tried and tested’ repeatedly to be accepted without question. Moreover, we opted to conduct a qualitative study to develop this conceptual model rather than a mixed methods study that would have included a quantitative component testing this model. This could not be carried out at this stage due to time and financial constraints but is planned as a next step to further improve and validate the model. At that stage, we may also need to revisit the themes that were discarded during this modified framework analysis process.

Finally, one of the objectives of the study was to gain insight into household and community level strategies. However, we were able to identify only those strategies that could change perceived societal response or influence societal norms, and assessed as feasible to deliver within the constraints of the main study. Other strategies such as, for example, pushcart vendors to supply fruits and vegetables and subsidies for low SES households, that require financial and personnel input and regular quality control from the *panchayat,* could not be included at this point.

## Conclusions

Through the ‘best-fit’ framework analysis, the integration of HBTs and qualitative findings resulted in development of a conceptual model that could facilitate the design of intervention strategies to aid a household-level dietary behaviour change process. The model includes *a priori* themes identified from the Trans-theoretical Model, the Health Belief Model and the Theory of Planned Behaviour that could be identified and indexed from the primary data. In addition, the model includes new themes, which emerged from the data that were not a part of the identified theories, but is essential to understanding the collective nature of this process. The findings of this study also underline the importance of developing dietary intervention strategies based on current practices in the households; the need to facilitate support mechanisms within the household through integration of other key members; and also remedy the gaps in awareness identified through this exercise. Lastly, it also emphasises the need to address socioeconomically disadvantaged families separately, through strong policy measures and strategic subsidies, to ensure an equitable expectation of success.

## Abbreviations

FGD: Focus group discussion; HBT: Health behaviour theories; TTM: Trans-Theoretical model; SCM: Social Cognitive model; HBM: Health Belief model; TPB: Theory of Planned Behaviour.

## Competing interests

The authors declare that they have no competing interests.

## Authors’ contributions

MD conceived and planned the study, developed the FGD and interview guide, collected and analysed the data, drafted and finalized the manuscript. RW participated in planning the study, development of the FGD and interview guide, data analysis, and critical revision and finalization of the manuscript. TKSR participated in planning of the study, analysis of data and finalization of the manuscript. KRT participated in planning of the study and critical revision of the manuscript. MR participated in data analysis and finalization of the manuscript. All authors read and approved the final manuscript*.*

## Pre-publication history

The pre-publication history for this paper can be accessed here:

http://www.biomedcentral.com/1471-2458/14/574/prepub

## Supplementary Material

Additional file 1 FGD and interview guide. Click here for file

Additional file 2 Definitions of the final themes mapped to construct the conceptual model. Click here for file

## References

[B1] Noar SM Zimmerman RS Health behavior theory and cumulative knowledge regarding health behaviors: are we moving in the right direction? Health Educ Res 2005 20 275 290 10.1093/her/cyg113 15632099

[B2] Painter JE Borba CP Hynes M Mays D Glanz K The use of theory in health behavior research from 2000 to 2005: a systematic review Ann Behav Med 2008 35 358 362 10.1007/s12160-008-9042-y 18633685

[B3] Daivadanam M Absetz P Sathish T Thankappan KR Fisher EB Philip NE Mathews E Oldenburg B Lifestyle change in Kerala, India: needs assessment and planning for a community-based diabetes prevention trial BMC Public Health 2013 13 95 10.1186/1471-2458-13-95 23375152 PMC3576354

[B4] Prochaska JO DiClemente CC Stages and processes of self-change of smoking: toward an integrative model of change J Consult Clin Psychol 1983 51 390 395 6863699 10.1037//0022-006x.51.3.390

[B5] Bandura A Health promotion by social cognitive means Health Educ Behav 2004 31 143 164 10.1177/1090198104263660 15090118

[B6] Horwath CC Applying the transtheoretical model to eating behaviour change: challenges and opportunities Nutr Res Rev 1999 12 281 317 10.1079/095442299108728965 19087455

[B7] Spencer L Wharton C Moyle S Adams T The transtheoretical model as applied to dietary behaviour and outcomes Nutr Res Rev 2007 20 46 73 10.1017/S0954422407747881 19079860

[B8] Povey R Conner M Sparks P James R Shepherd R A critical examination of the application of the Transtheoretical Model's stages of change to dietary behaviours Health Educ Res 1999 14 641 651 10.1093/her/14.5.641 10510072

[B9] Stokols D Translating social ecological theory into guidelines for community health promotion Am J Health Promot 1996 10 282 298 10.4278/0890-1171-10.4.282 10159709

[B10] Jabareen Y Building a conceptual framework: philosophy, definitions and procedure Int J Qual Meth 2009 8 49 62

[B11] Daivadanam M Wahlstrom R Ravindran TKS Sarma PS Sivasankaran S Thankappan KR Design and methodology of a community-based cluster-randomized controlled trial for dietary behaviour change in rural Kerala Glob Health Action 2013 6 20993 23866917 10.3402/gha.v6i0.20993 PMC3715653

[B12] Clark JP Godlee F, Jefferson T How to peer review a qualitative manuscript Peer Review in Health Sciences 2003 Second London: BMJ Books 219 235

[B13] Varatharajan D Thankappan R Jayapalan S Assessing the performance of primary health centres under decentralized government in Kerala, India Health Pol Plan 2004 19 41 51 10.1093/heapol/czh005 14679284

[B14] Fereday J Muir-Cochrane E Demonstrating rigor using thematic analysis: a hybrid approach of inductive and deductive coding and theme development Int J Qual Meth 2006 5 80 92

[B15] Dixon-Woods M Using framework-based synthesis for conducting reviews of qualitative studies BMC Med 2011 9 39 10.1186/1741-7015-9-39 21492447 PMC3095548

[B16] Carroll C Booth A Cooper K A worked example of "best fit" framework synthesis: a systematic review of views concerning the taking of some potential chemopreventive agents BMC Med Res Methodol 2011 11 29 10.1186/1471-2288-11-29 21410933 PMC3068987

[B17] Bryman A Burgess RG Analyzing qualitative data 1993 London & New York: Routledge

[B18] Strecher VJ Rosenstock IM Baum A The health belief model Cambridge Handbook of Psychology, Health and Medicine 1997 Cambridge, United Kingdom: Cambridge University Press 113 117

[B19] Ajzen I The theory of planned behavior Organ Behav Hum Decis Process 1991 50 179 211 10.1016/0749-5978(91)90020-T

[B20] United States Department of Health and Human Services Theory at a Glance. NIH Publication No. 05-3896 National Cancer Institute 2005 USA: National Institutes of Health

[B21] Berg-Smith SM Stevens VJ Brown KM Van Horn L Gernhofer N Peters E Greenberg R Snetselaar L Ahrens L Smith K A brief motivational intervention to improve dietary adherence in adolescents. The Dietary Intervention Study in Children (DISC) Research Group Health Educ Res 1999 14 399 410 10.1093/her/14.3.399 10539230

[B22] Shirazi M Assadi SM Sadeghi M Zeinaloo AA Kashani AS Arbabi M Alaedini F Lonka K Wahlstrom R Applying a modified Prochaska's model of readiness to change for general practitioners on depressive disorders in CME programmes: validation of tool J Eval Clin Pract 2007 13 298 302 10.1111/j.1365-2753.2006.00735.x 17378879

[B23] Michie S van Stralen MM West R The behaviour change wheel: a new method for characterising and designing behaviour change interventions Implement Sci 2011 6 42 10.1186/1748-5908-6-42 21513547 PMC3096582

[B24] Ahmed SM Petzold M Kabir ZN Tomson G Targeted intervention for the ultra poor in rural Bangladesh: Does it make any difference in their health-seeking behaviour? Soc Sci Med 2006 63 2899 2911 10.1016/j.socscimed.2006.07.024 16954049

