# Supplementary material for: Conceptual model for dietary behaviour change at household level: a ‘best-fit’ qualitative study using primary data
Source: BMC Public Health. 2014 Jun 9;14:574. doi: 10.1186/1471-2458-14-574 (PMC4080697; doi:10.1186/1471-2458-14-574)
Supplement: Additional file 1 — FGD and interview guide. [file 1471-2458-14-574-S1.pdf]

Additional file I

Guidelines: FGDs & individual Interviews

The guidelines for both the FGDs and the interviews were similar. While the FGDs covered general aspects of present day food habits in their respective communities and localities, the interviews focused on the specifics in particular households. The guidelines are given below:

I. General

1. Describe your family's food habits?
    a. How would you describe your food habits?
    b. In your opinion, would you describe them as healthy or unhealthy? Why?

II. Procurement, preparation and consumption

2. Fruits
    a. How frequently do you consume?
    b. What kind of fruits do you generally consume?
    c. What kind of fruits can you get from your neighbourhood?
    d. In what form do you consume fruits?
    e. How do you procure fruits in your household?
3. Vegetables
    a. How frequently do you consume?
    b. How do you usually take your vegetables? Only cooked? Also raw?
    c. If raw, which vegetables do you consume raw?
    d. What are the kinds of vegetables dishes that you make?
    e. Could you please describe how you cook vegetables?
4. Oil
    a. What oil do you use regularly?
    b. How do you procure your oil?
    c. Have you ever used any of the other oils?
    d. If yes, what was the experience like?
    e. If no, why?
    f. Any particular cooking practises in your household, like seasoning?

III. Food beliefs

5. Tell me about common fruits and vegetables available in your locality?
    a. Which would you consider as good? Why?
    b. Which would you not consider as good? Why?
6. Which rice do you usually use? White or red? Why?

IV. Availability and accessibility

7. Are fruits and vegetables readily available in you place?
8. Where can you purchase them from?
9. How much time does it take to get there?
10. How affordable are different fruits and vegetables?
11. Have you at any time decided not to purchase or procure particular fruits and vegetables? If so, what were the reasons?

V. Intervention and Strategies

12. Will the proposed study be of interest to your household and why? What could be the time frame to start making changes in your household?
13. Discuss the following strategies and how it can be modified.

Individual Level:

- Substitute snacks with fruits or veggies
- Eat a colourful plate: less rice and more vegetables
- Any suggestions for strategies at individual level

Household Level:

- Reallocate money used for snacks, beverages, bakery items or eating out
- Use drumstick leaves, bean leaves, keerai or others available around the house
- Make fruits and veggies visible and accessible around the house
- Any suggestions for strategies at household level

Community Level:

- Arranging push cart for supply of fruits and vegetables to inaccessible areas

- Growing of home garden by training households
- Cutting and selling vegetables
- Any suggestions for strategies at community level

VI. Informal assessment

14. How much of the following do you procure or consume in a month in your household?
    a. Salt
    b. Sugar, jaggery, jam and others
    c. Oil, ghee, and others
    d. Coconuts
