# Supplementary material for: Conceptual model for dietary behaviour change at household level: a ‘best-fit’ qualitative study using primary data
Source: BMC Public Health. 2014 Jun 9;14:574. doi: 10.1186/1471-2458-14-574 (PMC4080697; doi:10.1186/1471-2458-14-574)
Supplement: Additional file 2 — Definitions of the final themes mapped to construct the conceptual model. [file 1471-2458-14-574-S2.pdf]

Additional file 2

Definitions of the final themes mapped to construct the conceptual model

A priori themes after deductive reasoning

A priori themes retained as such

1. *Awareness:* The knowledge about health risks and the benefits of different health practices, including knowledge about the causes, consequences and solutions for a particular problem behaviour.
2. *Self-evaluation:* Self-assessment of a particular behaviour in terms of possibilities and potential avenues for bringing about a change in the said behaviour.
3. *Helpful relationships:* Includes relationships that promote caring, trust, openness, acceptance and support for the healthy behaviour change.
4. *Perceived seriousness:* Judgment as to the seriousness or severity of the disease, health problem or behaviour.
5. *Personal modifiers:* Characteristics that influence personal perceptions, such as culture, education level and past experiences.
6. *Attitude:* Refers to the degree to which a person has a favourable or unfavourable evaluation or appraisal of the behaviour in question.

A priori themes retained with modifications

1. *Identifying stages-of-change (pre-contemplation, intention and action):* This is the key construct of the model, which focused on identifying cues to differentiate households to three stages-of-change (instead of the original five: pre-contemplation, contemplation, preparation, action, maintenance).
    - Pre-contemplation (pre-contemplation): people do not intend to take action in the foreseeable future.
    - Intention (contemplation + preparation): people intend to take action in the immediate future, hence are more aware of the pros and cons; and have already taken some significant steps in a positive direction.
    - Action (action + maintenance): people have made specific overt modifications in their life-styles or are engaged to prevent relapse.
2. *Household efficacy:* belief or confidence in their ability as a household to control their behaviour and bring about a change or cope with different situations without relapsing to the earlier unhealthy habit.
3. *Decisional balance*: relative weighing of the pros and cons of changing behaviour; and their opinion of the value or usefulness of a new behaviour in decreasing the risk of developing a disease or health problem in terms of the expected costs and benefits.
4. *Substitution opportunities:* learning of healthier behaviour that can substitute for problem or unhealthy behaviour.
5. *Perceived risk:* self-assessment of the chances of acquiring a disease or health problem due to the continuation of an unhealthy behaviour.
6. *Perceived societal response:* perceived social pressure to perform or not to perform a particular behaviour.
7. *Cues to action:* events, people or things that prompt a desire to change behaviour.

New themes identified after inductive reasoning
1. *Accessibility:* combination of availability that includes physical access and affordability that includes all the costs associated with accessing foods.
2. *Perceived needs and preferences:* special needs and preferences of other household members as perceived by the female head of the household.
3. *Societal norms:* gender, socio-economic status and other higher hierarchical power structures in the household and community that influence behaviour.
4. *Perceived household response:* includes perceived willingness (or consent) to participate in a behaviour-change intervention; and the level of cooperation expected from other household members for such an endeavour as perceived by the female head of the household.
